# Is Antiretroviral Therapy Causing Long-Term Liver Damage? A Comparative Analysis of HIV-Mono-Infected and HIV/Hepatitis C Co-Infected Cohorts

**DOI:** 10.1371/journal.pone.0004517

**Published:** 2009-02-18

**Authors:** Erica E. M. Moodie, Nitika Pant Pai, Marina B. Klein

**Affiliations:** 1 Department of Epidemiology, Biostatistics, and Occupational Health, McGill University, Montreal, Quebec, Canada; 2 Divisions of Infectious Diseases/Immunodeficiency, Department of Medicine, Royal Victoria Hospital, McGill University Health Centre, Montreal, Quebec, Canada; National AIDS Research Institute, India

## Abstract

The effects of highly active antiretroviral therapy (HAART) on progression of hepatic fibrosis in HIV-hepatitis C virus (HCV) co-infection are not well understood. Deaths from liver diseases have risen in the post-HAART era, yet some cross-sectional studies have suggested that HAART use is associated with improved fibrosis rates. In a retrospective cohort of 533 HIV mono-infected and 127 HIV/HCV co-infected patients, followed between January 1991 and July 2005 at a university-based HIV clinic, we investigated the relationship between cumulative HAART exposure and hepatic fibrosis, as measured by the aspartate aminotransferase-to-platelet ratio index (APRI). We used a novel methodological approach to estimate the dose-response relationship of the effect of HAART exposure on APRI. HAART was associated with increasing APRI over time in HIV/HCV co-infected patients suggesting that they may be experiencing cumulative hepatotoxicity from antiretrovirals. The estimated median change (95% confidence interval) in APRI per one year of HAART intake was of −0.46% (−1.61% to 0.71%) in HIV mono-infected compared to 2.54% (−1.77% to 7.03%) in HIV/HCV co-infected patients. Similar results were found when the direct effect of HAART intake since the last visit was estimated on the change in APRI. HAART use associated is with increased APRI in patients with HIV/HCV co-infection. Therefore treatment for HCV infection may be required to slow the growing epidemic of end-stage liver disease in this population.

## Introduction

Since the advent of highly active antiretroviral therapy (HAART) there have been dramatic reductions of morbidity and mortality from virtually all causes among HIV-infected persons.[1], [2] One of the glaring exceptions to this trend is liver-related deaths which now represent one of the leading causes of death among HIV-infected individuals.[3]–[8] Much of this excess mortality has been driven by the epidemic of Hepatitis C (HCV) co-infection affecting more than 30% of HIV-infected patients in developed countries.[9], [10] HCV progresses more rapidly in patients co-infected with HIV.[11]–[13] In addition, non-alcoholic steato-hepatitis is increasingly being recognized as an important cause of liver disease in HIV-infected persons which may contribute to liver-related morbidity in the absence of HCV infection.[14]

A number of cross-sectional studies have suggested that HAART, especially regimens containing protease inhibitors (PI) [15]–[17], is associated with improved fibrosis rates. Other studies have observed no benefit.[18], [19] While it may be argued that increasing rates of liver outcomes is simply the “unmasking” of liver disease as individuals survive longer with HAART, other factors may be at play including irreversible hepatic damage, incomplete immune recovery, and chronic hepato-toxicity related to HAART. The net effect of HAART on liver disease therefore remains unclear.

The aspartate aminotransferase (AST) to platelet ratio index (APRI) has been validated as a surrogate marker of significant hepatic fibrosis in HIV/HCV co-infection.[20]–[22] We recently reported that the APRI was predictive of the development end-stage liver disease in HIV/HCV co-infection.[23] We used the APRI to evaluate the progression of liver disease in HIV-infected patients with and without HCV using standard linear regression models. We further evaluated the effect of HAART on liver disease progression and found that cumulative exposure, particularly to protease inhibitor-based regimens, was associated with increasing fibrosis in HIV/HCV co-infected patients and, to a lesser but nevertheless significant extent, in patients infected with HIV only.[23] For example, the estimated adjusted increase in slope of lnAPRI attributable to one year of HAART exposure was 0.18 vs. 0.07 units over 3 years in HIV/HCV co-infected and HIV-infected patients, respectively.

In this paper, we use an extension of propensity scores methodology to adjust for potentially confounding variables so as to evaluate whether HAART plays a role in liver disease progression in HIV-infected patients with and without HCV by modeling the dose-response function of APRI to HAART exposure.

## Methods

### Study design and setting

This is a retrospective cohort study from a university-based clinic serving approximately 1000 active HIV-infected patients. Since 1989, a computerized database on all patients has been maintained into which demographic data, clinical diagnoses (both AIDS and non-AIDS), laboratory and prescription information are prospectively entered. Any changes in antiretroviral medications are reviewed at each clinic visit by a physician and/or clinic pharmacist and recorded. This study was approved by the Institutional Research Ethics Board. As this was a retrospective review of electronic records, we did not obtain informed consent from study participants, in accordance with regulations of our intuitional ethics review board which approved the study.

All participants were HIV sero-positive (determined by positive ELISA with confirmatory Western blot), attended the clinic at least twice between January 1991 and December 2004, and did not have a clinical diagnosis of cirrhosis at baseline. Subjects were categorized by HIV/HCV co-infection or by HIV infection alone. Patients with both HCV and chronic hepatitis B virus infections were excluded. HCV testing was performed using ELISA followed by confirmatory testing of positive samples with recombinant immuno-blot assays. Patients were followed until July 2005 or censored on their last clinic visit if lost to follow-up. Ten patients were censored when they received specific HCV therapy and a further 13 were excluded from the analysis due to missing information at each visit on at least one variable of interest. The analyzed cohort was thus comprised of 660 subjects monitored over 14 years: 533 who were HIV infected and 127 who were co-infected with HCV.

Baseline demographic, clinical and laboratory variables, as well as follow-up data for 6-month intervals, were extracted from the database. Concurrent measures (taken not more than 7 days apart) of AST and platelets were used to calculate APRI values. For each 6-month interval the most recent APRI value was used. CD4+ T-cell counts were measured by flow cytometry. Plasma HIV viral load measurements were performed using Chiron branched chain DNA assays (standardized to Quantiplex version 3.0 equivalent as previously described[24]).

### Exposure and covariate assessment

The exposure of interest was HAART intake, calculated as the total number of years on HAART up to the last APRI measurement in each interval. HAART was defined as at least three antiretrovirals taken concurrently for more than one day. Triple nucleoside regimens were considered as HAART if they contained abacavir. We considered the following variables that may confound the relationship between HAART and liver function: age, gender, region of birth, transmission risk factors (men who have sex with men (MSM) and injection drug use), calendar year at study entry, use of HAART prior to baseline, CD4+ T-cell counts, CD8+ T-cell counts, and plasma HIV RNA (log copies/ml). CD4 and CD8 counts and HIV RNA were measured at follow-up visits and were therefore time-varying.

### Outcome assessment

**Figure 1:**
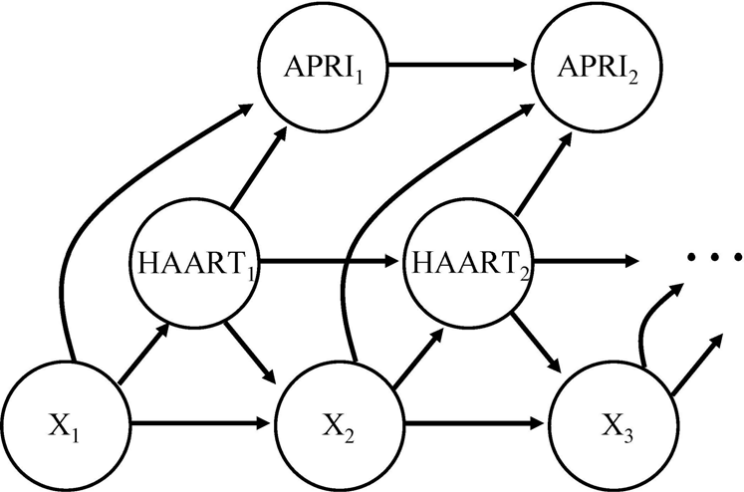
Postulated causal diagram. A directed acyclic graph of the relationship between HAART exposure and APRI over time, where potentially confounding variables such as, for example, CD4+ T-cell counts, are represented by X. The Generalized Propensity Score permits unbiased estimation of the direct (or cross-sectional) effect, α, of most recent HAART intake on the response.

The outcome of interest was the natural logarithm of the APRI (lnAPRI). The APRI was defined as: 100×(AST/upper limit of normal)/platelet count (10^9^/l).[25] An APRI score>1.5 has been shown to be predictive of significant fibrosis (defined as an Ishak score greater than 3) with an the area under the ROC of 0.76–0.85.[21], [22], [25] Thus, we used the established cutoff of 1.5 to determine the presence of significant fibrosis at baseline and at the last visit. Similarly, an APRI<0.5 has been shown to rule out the presence of fibrosis[25] and was used to define its absence. The log of the APRI, a transformation which nearly normalizes the distribution, was used in all analyses however results are reported for APRI to facilitate clinical interpretation. Baseline was defined as the date of the first APRI measure, which generally occurred within one month of the first clinic visit. The response, lnAPRI, is not a health outcome typically considered by treating physicians and is not used in clinical practice to inform treatment decisions with respect to HAART; see Figure 1 for the postulated causal diagram.

### Statistical methods

We focused on the relationship between HAART and liver function. We began by examining change in lnAPRI between clinic visits caused by the dose of HAART received in that time, that is, we examined the direct or “cross-sectional” effect of most recent HAART intake on the change in APRI (see Figure 1). This gave rise to a longitudinal data model that assumes a repeated measures structure, where each participant provided a time-series of measurements both for the outcome, change in lnAPRI, for the exposure of interest, HAART intake since the last visit, and for time-varying covariates, i.e., CD4+ T-lymphocyte count, CD8+ T-lymphocyte count, and HIV RNA. We used an extension of the propensity scores approach, the Generalized Propensity Score (GPS)[26]–[28] that has been broadened to the repeated measures data scenario (as described in *Estimation of Dose-Response Functions for Longitudinal Data using The Generalized Propensity Score* by EEM Moodie and DA Stephen (2009), manuscript in revision) to estimate the marginal dose-response curve to model the effect of HAART on liver function as measured by the lnAPRI. For details, see Appendix S1.

As not all subjects had used HAART at all points in the study, the predictive model for treatment, i.e. the GPS, needed to acknowledge that the nature of the dose distribution was a mixture of a mass at zero dose and a continuous distribution. Estimation of the density of cumulative HAART is straightforward if a parametric form for the continuous part of the distribution is assumed. We used a Box-Cox transformation of positive cumulative doses with power parameter selected by maximum likelihood to render the data more approximately normal.[29] The transformed doses were modeled under a normal density whose mean was a linear function of age, sex, region of birth, year of study entry, use of HAART prior to baseline, time in cohort, co-infection status, injection drug use, MSM, CD4+ T-cell counts, CD8+ T-cell counts, and HIV RNA, as well as interactions between co-infection status and each of CD4+ T-cell count, HIV RNA, injection drug use, and MSM. To estimate the probability of having a non-zero dose of cumulative HAART in any interval, a logistic model to the binary (HAART = 0, HAART>0) dose data was used, including the same predictors are in the continuous-dose portion of the mixture distribution. We further explored the relationship between HAART and liver function by examining the lnAPRI at each clinic visits as a function of the dose of HAART received up to that time.

### Secondary analyses

To explore differences between PI-based HAART and non-nucleoside reverse transcriptase inhibitors (NNRTI)-based HAART, two additional GPS analyses were conducted. In addition to the variables included in the main analyses (see above), we also included cumulative NNRTI use in the analysis of the effects of PI on APRI, and similarly included cumulative PI use when analyzing the effects of NNRTI on APRI. We also considered an analysis in which attention was restricted only to patients without significant fibrosis at baseline (i.e. APRI<1.5).

## Results

### Baseline characteristics of study subjects

The cohort contained information from 4496 visits (3718 from mono-infected and 778 from co-infected patients) with complete information on CD4+ T-cell counts, CD8+ T-cell count, HIV RNA, and lnAPRI from 660 HIV patients. A total of 533 patients were infected with HIV alone and 127 were co-infected with HCV. A non-negligible proportion of the cumulative HAART intake measurements (906/4496 or 20%) reported no intake.

The duration of follow-up was comparable between the mono- and co-infected groups. Baseline characteristics of the subjects are shown in Table 1. Mean CD4+ T-cell counts were higher in co-infected than mono-infected patients and, on average, CD8+ T-cell counts were higher and viral loads were lower in patients with HIV alone. The most striking difference between the two patient groups was history of MSM, which was more frequent in the mono-infected group, and of injection drug use, which was predominant in the co-infected patients. The majority of the cohort in both patient populations was born in Canada; however, the proportion was much higher in the co-infected (83.5%) than mono-infected (58.0%) patients.

**Table 1:** Baseline characteristics according viral co-infection status.

		HIV mono-infected (n = 533)	HIV/HCV co-infected (n = 127)
**Length of follow-up**	(months)	36.3 (0, 169.4)	31.8 (0, 146.3)
**Number of visits**	1–5	261 (50.0)	67 (52.8)
	6–10	149 (28.0)	39 (30.7)
	>10	123 (22.0)	21 (18.5)
**Age**	(years)	37.7 (18, 72.7)	37.4 (19.5, 63.5)
**Sex**	(male)	396 (74.3)	94 (74.0)
**Region of birth**	Africa	94 (17.6)	7 (5.5)
	Asia	6 (1.1)	2 (1.6)
	Canada	309 (58.0)	106 (83.5)
	Europe	29 (5.4)	9 (7.1)
	Haiti	71 (13.3)	1 (0.8)
	Latin America/Caribbean	24 (4.4)	2 (1.6)
**Intravenous drug use**		12 (2.3)	94 (74.0)
**Males with history of sex with men**		281 (52.7)	14 (11.0)
**Year of study entry**	1991–1995	91 (17.1)	19 (15.0)
	1996–2000	217 (34.1)	61 (41.7)
	2000–2004	225 (48.8)	47 (43.3)
**CD4+ T-cell count**	(×10^9^ cells/ml)	227 (0, 1753)	290 (2, 1017)
**CD8+ T-cell count**	(×10^9^ cells/ml)	706 (0, 4919)	600 (0, 2190)
**Plasma HIV RNA**	(log copies/ml)	3.98 (1.70, 6.59)	4.19 (1.70, 6.09)
**APRI**		0.31 (0.05, 11.43)	0.59 (0.13, 20.85)
**Prior use of HAART**		182 (34.1)	39 (30.7)

For continuous variables, statistics reported are median (range). For categorical variables statistics reported are count (%).

Abbreviations: APRI, AST to platelet ratio index; HAART, highly active antiretroviral therapy; HCV, hepatitis C infection.

The baseline prevalence of significant fibrosis (APRI>1.5) was 3.9% (21/533) in HIV mono-infected patients and 17.3% (22/127) of HIV/HCV co-infected patients. The proportions of patients with APRI<0.5 at baseline were 77.7% (414/533) of HIV mono-infected patients and 43.3% (55/127) of HIV/HCV co-infected patients.

### The direct effect of HAART use on liver disease progression

**Figure 2:**
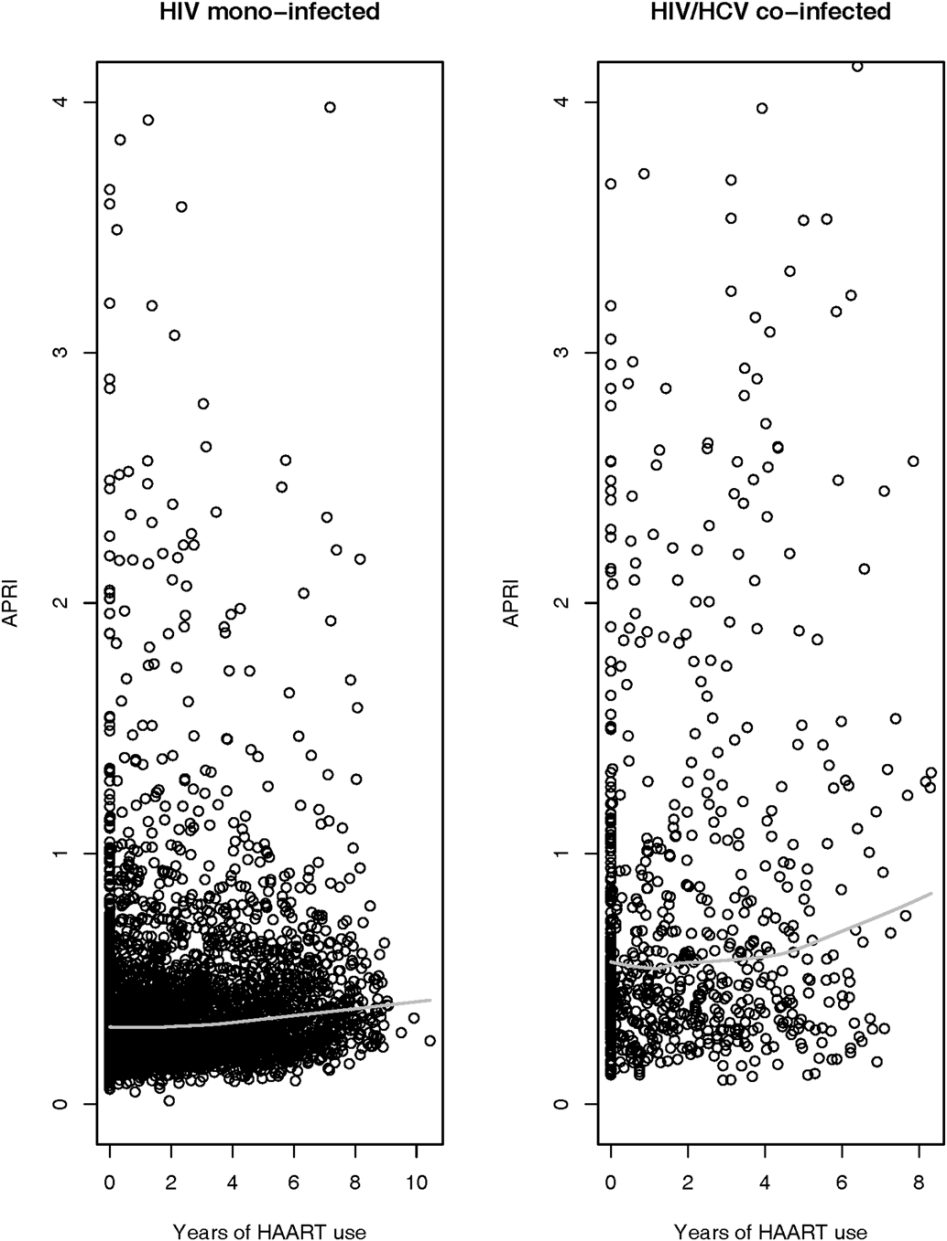
APRI by cumulative HAART exposure in years over the course of the study. The figure on the left is HIV-infected and on the right is the HIV/HCV co-infected patients; 15 data points in mono-infected persons and 39 points in co-infected persons have APRI scores above 4.0.

Exploratory plots of lnAPRI by cumulative HAART intake suggested a positive relationship (Figure 2). The GPS and time in the cohort not on HAART were included in a linear mixed model with cumulative HAART, which also allowed for modification of the effect of HAART by co-infection status.

**Figure 3:**
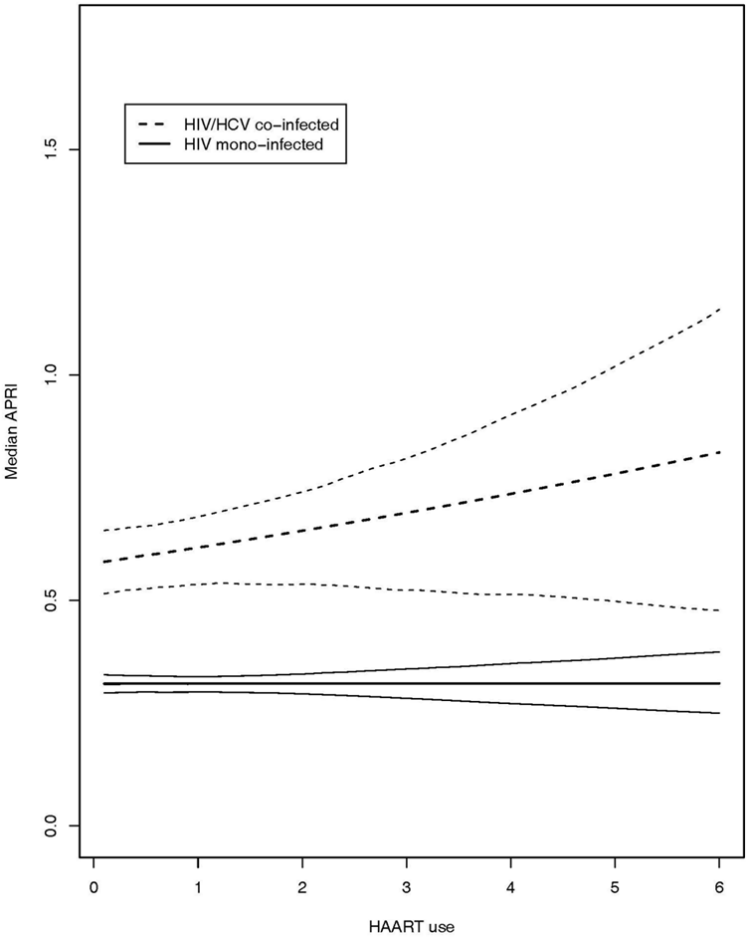
Estimated dose-response function: change in APRI due to HAART intake between clinical visits. The plot includes point-wise 95% confidence bands for HIV mono-infected patients (solid line) and HIV/HCV co-infected patients (dashed line).

Analyses of the effect of HAART consumed since last clinic visit on liver fibrosis progression was performed in a subset of the full cohort (subjects were required to have at least two clinic visits for changes in dose and lnAPRI measurements to be recorded); baseline characteristics are summarized in Table 2. The estimated causal dose-response curve was lower than we previously reported[23]; in fact, no significant change in APRI was found to be caused by HAART intake in people infected with HIV alone and only a small increase was observed in co-infected individuals (Figure 3). The response to dose since the last clinic visit found no significant change in APRI caused by HAART intake in people infected with HIV alone. An increase was observed in co-infected individuals (Figure 3). Among mono-infected persons, the median increase in APRI attributable to one year of HAART intake was approximately 0.04% (95% CI: −3.88 to 4.12) compared to 6.05% (95% CI: −1.82 to 14.15) in co-infected populations.

**Table 2:** Baseline characteristics according viral co-infection status (individuals used to assess the by-interval effects).

		HIV mono-infected (n = 533)	HIV/HCV co-infected (n = 127)
**Length of follow-up**	(months)	43.4 (1.6, 169.4)	38.3 (0.9, 146.3)
**Number of visits**	1–5	296 (56.7)	66 (55.5)
	6–10	130 (24.9)	37 (31.1)
	>10	96 (18.4)	16 (13.4)
**Age**	(years)	37.7 (18.1, 72.7)	37.6 (19.5, 63.5)
**Sex**	(male)	389 (74.5)	89 (74.8)
**Region of birth**	Africa	91 (17.4)	7 (5.9)
	Asia	6 (1.1)	2 (1.7)
	Canada	305 (58.4)	99 (83.2)
	Europe	28 (5.4)	9 (7.6)
	Haiti	68 (1303)	1 (0.8)
	Latin America/Caribbean	24 (4.6)	1 (0.8)
**Intravenous drug use**		12 (2.3)	89 (74.8)
**Males with history of sex with men**		276 (52.9)	13 (10.9)
**Year of study entry**	1991–1995	86 (16.5)	16 (13.4)
	1996–2000	214 (41.0)	57 (47.9)
	2000–2004	222 (42.5)	46 (38.7)
**CD4+ T-cell count**	(×10^9^ cells/ml)		
**CD8+ T-cell count**	(×10^9^ cells/ml)	728 (0, 4141)	592 (0, 1845)
**Plasma HIV RNA**	(log copies/ml)	3.18 (1.70, 6.00)	3.18 (1.70, 5. 29)
**APRI**		0.30 (0.08, 6.84)	0.57 (0.12, 19. 65)
**Prior use of HAART**		179 (34.13)	38 (31.9)

For continuous variables, statistics reported are median (range). For categorical variables statistics reported are count (%).

Abbreviations: APRI, AST to platelet ratio index; HAART, highly active antiretroviral therapy; HCV, hepatitis C infection.

### Relating cumulative HAART use to liver disease progression

**Figure 4:**
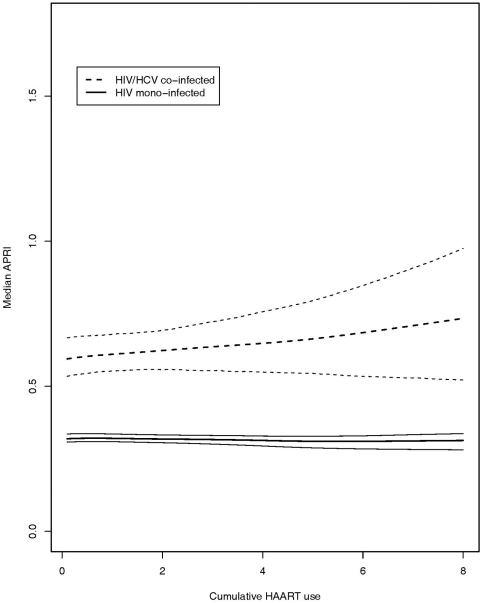
Estimated dose-response function: APRI due to cumulative HAART intake. The plot includes point-wise 95% confidence bands for HIV mono-infected patients (solid line) and HIV/HCV co-infected patients (dashed line).

Patterns of APRI response to cumulative HAART were similar to those found in the estimation of the direct of effects of most recent dose on changes in APRI (Figure 4). Among mono-infected persons, the median increase in APRI attributable to one year of HAART intake was approximately −0.46% (95% CI: −1.61% to 0.71%) compared to 2.54% (95% CI: −1.77% to 7.03%) in co-infected populations. Thus, over five years we would expect to see an increase in the median APRI of approximately 13.35% (95% CI: −8.52% to 140.47%) in HIV/HCV-infected populations. Unsurprisingly, the median APRI of the mono- and co-infected individuals differed considerably in the absence of HAART use: estimated APRI under no intake of HAART was 0.32 (95% CI: 0.31 to 0.34) in the HIV-only cohort and 0.54 (95% CI: 0.53 to 0.55) in the HIV/HCV-infected group.

### Secondary analyses

Several additional analyses were undertaken to further explore the effect of HAART on APRI. We explored of the effects of cumulative PI and NNRTI on APRI. The median increase in APRI per one year of PI intake was 0.47% (95% CI: −0.99% to 1.86%) in HIV mono-infected as compared to 3.31% (95% CI: −2.09% to 9.02%) in HIV/HCV co-infected patients. The median increase in APRI per one year of NNRTI intake was 0.55% (95% CI: −1.26% to 2.38%) in HIV mono-infected as compared to 2.18% (95% CI: −6.53% to 11.69%) in HIV/HCV co-infected patients. This confirms the previous results which suggested that increased APRI on HAART may be more attributable to PI use than NNRTI use.

The sub-group analysis focusing on patients without significant fibrosis at baseline was performed on 4242 observations in 647 individuals. The median increase in APRI per one year of HAART intake was 0.48% (95% CI: −0.61% to 1.59%) in HIV mono-infected as compared to 3.44% (95% CI: −0.92% to 7.98%) in HIV/HCV co-infected patients, indicating that the relationship observed between HAART and APRI is not driven by the sub-set of patients with significant fibrosis at the start of the study.

## Discussion

To date, the study of the effects of HAART on liver fibrosis over time has been limited primarily to mathematical models based on single measurements of liver fibrosis. No previous study has focused on repeated balancing of patient covariates over time in order to estimate dose-response curves.

Our study used a large clinical cohort with data collection and follow-up spanning over a decade. With the availability of detailed clinical and laboratory information, we were able to adjust for many potential baseline and follow-up confounders. In addition, care was taken to ensure that the impact of time, both in terms of the duration of HIV infection and calendar time, was taken into account in the analysis. We have thus shown that HAART appears to have a negligible effect on liver fibrosis progression as measured by the APRI in HIV-infected patients, but is associated with an increase in fibrosis progression in HIV/HCV co-infected individuals. This finding contrasts somewhat with our previous results obtained using standard mixed models where we observed that HAART was associated with fibrosis progression in both groups.[23]

### Potential limitations of the present study

As with all models for observational data, approaches such as the GPS require assumptions to be appropriately specified.[30], [31] The GPS approach has been shown to be a powerful tool in the analysis of direct effects of continuous dose in cross-sectional[28] and longitudinal data (*Estimation of Dose-Response Functions for Longitudinal Data using The Generalized Propensity Score* by EEM Moodie and DA Stephen (2009)) however the methodology does not control for unmeasured confounding variables. Diagnostic plots (not shown) suggested that the constructed GPS appropriately broke the confounding relationships for those variables that were recorded. It is nevertheless possible that estimates are confounded by factors for which data were unavailable such as alcohol use, the presence of other infections, and the use of hepato-toxic medications. We surmise that patients who use alcohol heavily are less likely to maintain a HAART regimen than patients who do not consume alcohol. Under this assumption, our results likely underestimate the increase in APRI caused by HAART use. Future work will include an analysis of a prospective co-infection cohort on whom alcohol intake information is available.

A further complication of longitudinal data such as these is that time-varying variables such as CD4 cell counts could potentially act as mediating variables in addition to being confounding variables. In such cases, when the total (direct and indirect) effect of cumulative dose is to be estimated, the use of marginal structural models is typically preferred over adjusting for such variables in a regression model, which is, in effect, the result of the GPS approach. However in the present study, the highly varied number of visits per individual in the study (recall that approximately 50% of individuals present for five visits or fewer while one fifth of the participants present to more than 10) and the absence of a biologically meaningful baseline time make the marginal structural model approach less appealing. Furthermore, the implementation of the inverse weighting required for marginal structural models is not straightforward when dose densities are mixtures distributions with continuous components, as in the present study.

We therefore proceeded with the GPS analysis, and were reassured by the concordance of conclusions between the estimation of the direct effect of HAART intake since the last visit on change in APRI and the analysis that examined the effects of cumulative HAART on APRI. We note that the primary analysis which investigated the effect of HAART received between two clinic visits on the change in APRI between the two visits after intake of that HAART does in fact provide an estimate of causal parameter. However in the analysis in which we considered the effect of cumulative HAART on APRI, the relationship estimated captures a combination of the effect of the most recent dose of HAART and additionally the effect of HAART consumed at previous intervals that is not mediated through the time-varying confounding variables. We suspect that this leads to an underestimate of the total effect of cumulative HAART intake on liver function, however this relationship is difficult to surmise as the interactions between immunological factors such as lymphocyte counts and viral load on the components of the APRI are not fully understood.

Another potential limitation of our study was the use of APRI as a surrogate marker for liver fibrosis. Both AST and platelets may be subject to variations due to factors other than liver disease progression. Ideally, the evolution of hepatic fibrosis would be assessed by multiple liver biopsies in a large cohort. However, the acceptability, cost, and risk associated with multiple biopsies make such a study impractical. Liver biopsy itself is an imperfect measure as it is subject to high sampling variability[32] and considerable intra- and inter-observer variability in the histological assessment of the biopsy.[33] Therefore, in the absence of even a perfect invasive reference standard, non-invasive markers are more appealing to clinicians and patients alike. Non-invasive measures of liver fibrosis are gaining acceptance for long-term evaluation of hepatic complications in HCV co-infection.[34] The APRI compares favorably with other non-invasive biomarkers such as the FIB-4[35], the MULTIVIRC equation[36], and the Forns index[37] that do not incorporate the same parameters.

### Conclusions

We found that the APRI was not increased by long-term HAART use in patients infected with HIV alone, but was associated with increased APRI in patients with HIV/HCV co-infection. As HAART appears to increase fibrosis progression in HIV/HCV co-infected patients possibly through cumulative hepato-toxicity, specific treatment for HCV infection will be required to slow the growing epidemic of end-stage liver disease in this population.

## Supporting Information

Appendix S1 The Multivariate GPS Fitting Procedure
(0.04 MB DOC)
