# Supplementary material for: Is Antiretroviral Therapy Causing Long-Term Liver Damage? A Comparative Analysis of HIV-Mono-Infected and HIV/Hepatitis C Co-Infected Cohorts
Source: PLoS One. 2009 Feb 18;4(2):e4517. doi: 10.1371/journal.pone.0004517 (PMC2637977; doi:10.1371/journal.pone.0004517)
Supplement: Appendix S1 — The Multivariate GPS Fitting Procedure (0.04 MB DOC) [file pone.0004517.s001.doc]

**Appendix S1: The Multivariate GPS Fitting Procedure**

Suppose that data are collected on *N* individuals over time, so that *n*_i_, i = 0,...,*N* measurements are available. Thus, each individual is observed for *N* intervals, with the initial or baseline measurement taken at time j=0. For individual i at measurement time j, denote the response by Y_ij_, the treatment dose taken to that time by D_ij_, and other potentially confounding covariates by X_ij_; observed values of the random variables will be indicated by y, d, and x respectively. The analysis is formulated through the use of potential outcomes: the value of the outcome that would result at time j if an individual were to receive a specified dose which may not equal the dose actually received in the study. We denote by Y_ij_(d) the random variable resulting from a dose D_ij_ = d taken in interval j. At j=0, D_i0_ is typically set to zero, with any doses taken prior to the study coded as a baseline covariate.

Define the observed Multivariate Generalized Propensity Score, r_ij_(d,x) for dose d and covariate values x by

r_ij_(d,x) = f(d_ij_|X_ij_=x)

that is, the conditional density function for dose D at time j given covariates X_ij_=x evaluated at D_ij_=d.(29) This is an extension of the (single-time) Generalized Propensity Score(26-28), which extended the usual propensity score for binary treatments to multi-category or continuous treatments. The Multivariate GPS random quantity R_ij_ acts as a balancing score: D_ij_ and X_ij_ are conditionally independent given R_ij_ and the distribution of the potential response at time j is independent of the covariates measured to that time, given the treatment and the Multivariate GPS. Consequently, controlling for R_ij_ provides (conditional) independence between D_ij_ and X_ij_, while the conditional independence of Y_ij_ and the covariates given treatment dose and the Multivariate GPS simplifies the response model construction.

The causal dose-response function is found by considering the average dose-response curve at dose level d, μ(d) = E[Y_ij_(d)] = E{[E[Y_ij_(d)|X_ij_]}, which traces the causal dose-response relationship as d varies in its support. The multivariate estimation procedure, an adaptation of the one-interval procedure(26) to a repeated measures scenario, proceeds as follows:

1. Form the MGPS model using a regression approach by constructing a predictive model, f(d|x,*β*), for D_ij_ given X_ij_. Estimate the parameters *β* using the observed data.
2. Compute the fitted Multivariate GPS values as r_ij_ = f(d|x,$\hat{\beta}$).
3. Form the mean response model using a regression approach by constructing a predictive model, f(y|d,r,*α*), for Y_ij_ given D_ij_ and R_ij_. For example, a random effects model with random intercept to account for within-person variability may be used. Estimate parameters α using the observed data and the fitted Multivariate GPS values of the previous step.
4. Estimate the dose-response curve at dose level d by

$\hat{\mu}$(d) = $\frac{1}{N}\sum_{i=i}^{N} \sum_{j=1}^{n_{i}}$E[Y_ij_(d) | D_ij_=d, R_ij_=r(d,x_ij_),$\hat{\alpha}$]

for d in a suitable range.

The Multivariate GPS approach described above assumes a common dose-response relationship over time. It is equally possible to include the time variable, j, and an interaction between time and dose level D_ij_, in the predictive model provided time on study is an exogenous variable.

Much like the usual propensity score approach, the Generalized Propensity Score (multivariate or otherwise) provides a mechanism to ensure and assess balance of the doses across the measured covariates, which could otherwise be a difficult task in the presence of many covariates. Furthermore, rather than attempting to specify a form for the response as a function of dose and potentially many other confounding variables, the GPS procedure simplifies fitting procedures by breaking this into two steps, first modelling the treatment or dosing mechanism and then modelling the response very simply, including only the dose and the estimated GPS as predictors.
